# Ocular surface toxicity of depatuxizumab mafoditin (ABT-414): case reports

**DOI:** 10.5935/0004-2749.20220039

**Published:** 2025-08-21

**Authors:** Carlos Rocha-de-Lossada, Carmen Alba Linero, Álvaro Santos Ortega, Marina Rodríguez Calvo-de-Mora, Rahul Rachwani, Davide Borroni, Emilio Alba, Manuel Benavides Orgaz, Vito Romano

**Affiliations:** 1 Ophthalmology Department, Hospital Regional Universitario de Málaga, Málaga, Spain; 2 Royal Liverpool University Hospital, Liverpool, United Kingdom; 3 Department of Doctoral Studies, Riga Stradins University, Latvia; 4 Unidad de gestión clínica, Oncología Médica Hospital Regional y Universitario de Málaga. Instituto de Investigación de Biotecnología de Málaga, Málaga, Spain

**Keywords:** Glioblastoma/drug therapy, Antibodies, monoclonal, humanized/therapeutic use, Cornea/drug effects, Visual disorders/etiology, Humans, Case reports, Glioblastoma/tratamento farmacológico, Anticorpos monoclonais humanizados/uso terapêutico, Córnea/efeitos de fár macos, Transtorno da visão, Humanos, Relato de caso

## Abstract

The purpose of this study is to report the clinical features and outcomes of ocular surface toxicity following depatuxizumab mafoditin (ABT-414) therapy for unresectable glioblastoma. Ocular signs and symptoms of three patients treated with ABT-414 during a phase III trial for glioblastoma multiforme were evaluated. Both eyes of all patients were damaged during the week after the first infusion of the ABT-414 molecule. In all patients, mild-to-moderate keratitis could be ascertained, along with decreased visual acuity and blurred vision, as well as foreign-body sensation and redness. Symptoms and visual acuity improved 4 weeks. In conclusion, ABT-414 therapy may cause transient ocular surface toxicity. The initiation of artificial tears and lubricant ointment was enough to control the ocular surface signs and symptoms. A multidisciplinary approach, complete ophthalmologic monitorization, and elaboration of protocols are required to adequately manage these patients.

## Abstract (pt)

Nosso objetivo é relatar as características clínicas e os resultados da toxicidade na superfície ocular após a terapia com depatuxizumabe mafodotina (ABT-414) para glioblastoma irressecável. Os sinais e sintomas oculares de três pacientes que foram tratados com ABT-414 durante um estudo de fase III para glioblastoma multiforme foram avaliados. Ambos os olhos de todos os pacientes foram danificados durante a semana após a primeira infusão da molécula ABT-414. Em todos os pacientes, uma ceratite de leve a moderada pode ser verificada, juntamente com uma diminuição da acuidade visual e visão turva, bem como sensação de corpo estranho e vermelhidão. Os sintomas e a acuidade visual melhoraram em um período de 4 semanas. Em conclusão, a terapia com ABT-414 pode causar toxicidade transitória na superfície ocular. A iniciação com lágrimas artificiais e pomada lubrificante foi suficiente para controlar os sinais e sintomas na superfície ocular. Uma abordagem multidisciplinar, com acompanhamento oftalmológico completo e a elaboração de protocolos são necessários para o manejo adequado desses pacientes.

## INTRODUCTION

Depatuxizumab mafoditin, also known as ABT-414, is an antibody drug conjugate designed to treat tumors harboring amplified genomic epidermal growth factor receptor (EGFR)^(1)^.

ABT-414 is a newer-generation antibody drug conjugate that consists of a veneered humanized recombinant IgG1κ antibody with binding properties specific to a unique epitope of human EGFR. It also consists of noncleavable maleimido-caproyl linkers, each of which is attached to a potent antimicrotubule agent, monomethylauristatin F (MMAF)^(1)^.

Glioblastoma (GB) is regarded as the most common malignant brain tumor in adults, accounting for 47.1% of all malignant brain tumors^(2)^.

Previous studies have described the ocular side effects of EGFR-blocking antibody therapy used in other neoplasms^(3)^. Some authors have already described the occurrence of ocular surface and corneal toxicity after MMAF associated with EGFR inhibitor treatment, including conjunctival hyperemia, intraepithelial cysts, stromal edema, superficial punctate epitheliopathy, and blepharitis^(4)^. Moreover, confocal microscopy studies have revealed corneal changes, such as diffuse hyperreflective white round spots in the corneal basal epithelial layers and subbasal nerve plexus layer fiber fragmentation^(5)^.

In this case series, we describe short-term painless ocular surface toxicity in three patients treated with ABT-414.

## CASE SERIES

In this case series, consisting of a descriptive prospective analysis, we examined six eyes of three patients treated with depatuxizumab mafoditin (ABT-414) for GB. Examinations were carried out in the Ophthalmic Department of Hospital Regional Universitario of Málaga, Spain. All patients were treated with 0.1% dexamethasone phosphate solution four times a day for 7 days, starting 48 hours before each infusion of the ABT-414 molecule, as indicated by the clinical trial protocol^(1)^. All patients underwent a complete ophthalmologic examination, consisting of intraocular pressure (IOP) measurement, tear film breakup time (BUT), Schirmer test, and corneal and conjunctival staining using fluorescein dye (according to the Oxford Grading Scale). To evaluate ocular pain, patients were asked to rate their average severity of ocular pain using a 4-point scale including *no pain* (1), *mild pain* (2), *moderate pain* (3), and *most severe pain* (4). To evaluate corneal sensitivity, we used a cotton bud tip to stimulate the corneal pain response.

The mean age of the patients was 48.64 ± 8.97 years; two were female. The day before the first infusion, the mean best-corrected visual acuity (BCVA) was 20/20 (Snellen) in all patients, and the ocular surface examination was unremarkable. The Mean Schirmer test value was 12.1 ± 1.4 mm, and the mean BUT was 10.7 ± 2.9 seconds. The mean IOP value was 15.4 ± 3.2 mmHg. One week after the infusion, patients complained of foreign-body sensation (n=3), redness (n=3), blurred vision (n=3), and photophobia (n=2). Despite the presence of other symptoms, ocular pain was reported in only one subject, and it was reported as mild (score 1).

**Figure 1:**
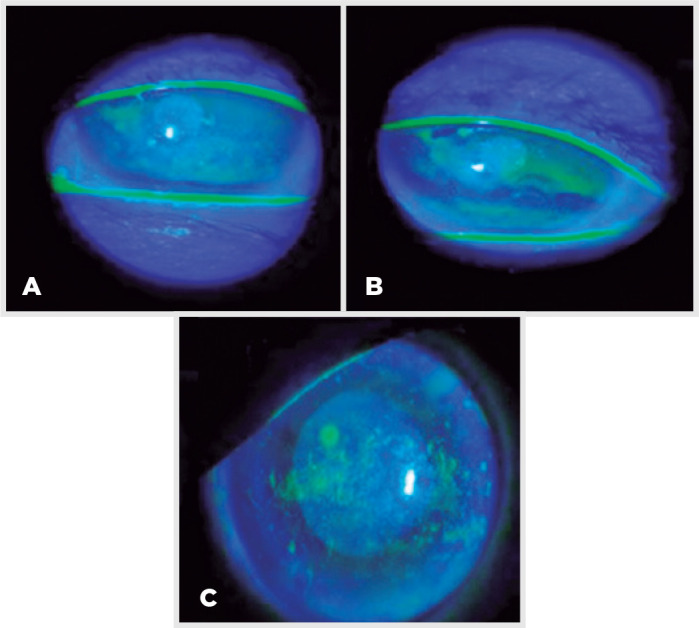
(A, B, C) Slit lamp examination (fluorescein staining) showing diffuse punctate keratitis and corneal microcysts in patients treated with depatuxizumab mafoditin (ABT-414).

Epithelial keratitis was evaluated using the Oxford scale (range, 0-IV; Figure 1). All eyes presented as grade ≥II on the Oxford scale, except for one eye, which presented as grade II. We also observed irritative conjunctivitis and signs of anterior blepharitis, such as hyperemia and telangiectasias, on the eyelid margin in all subjects. The dilated fundus examination remained unremarkable. Table 1 presents the values of BCVA, Schirmer test, Oxford scale, and BUT 1 week after infusion. All patients were treated with preservative-free topical artificial tears. As the ocular side effects were mild and managed satisfactorily, it was decided jointly with the oncology department not to suspend treatment with ABT-414, because of the risk-benefit in the survival of these patients. In all patients, symptoms and visual acuity improved over a period of 4 weeks.

**Table 1:** Examination values 1 week after ABT-414 molecule infusion

Patient	BVCA (Snellen)		IOP (mmHg)		Schirmer I test (mm)		BUT (s)		Oxford scale	
	RE	LE	RE	LE	RE	LE	RE	LE	RE	LE
**1**	20/40 ± 20/100	20/32 ± 20/100	12.8 ± 1.9	12 ± 2	12 ± 0.2	12.2 ± 0.3	11.1 ± 0.6	11.4 ± 0.8	II-III	II-III
**2**	20/40 ± 20/100	20/40 ± 20/100	10.5 ± 1.5	11.8 ± 2.1	11.7 ± 0.4	12.5 ± 0.2	10 ± 0.6	11.3 ± 0.2	II	III
**3**	20/25 ± 20/100	20/32 ± 20/100	16 ± 2	15.4 ± 1.7	13.4 ± 0.2	12.7 ± 0.3	12.2 ± 0.4	11.3 ± 0.5	II-III	III

BCVA= best-corrected visual acuity; IOP= intraocular pressure; BUT= breakup time; RE= right eye; LE= left eye.

## DISCUSSION

GB is the most frequent malignant brain tumor, and it is often aggressive and unresectable^(6)^. to extend the survival time of patients with GB, adjuvant and concomitant therapies have been developed in recent years. We present three cases of GB treated with ABT-414, which is a newer-generation antibody drug conjugate used for the treatment of GB.

The severity of the adverse events should be graded according to the Common Terminology Criteria for Adverse Events scale^(7)^. Preclinical studies have shown that ABT-414 is related to systemic adverse events, such as allergic reactions or dermatologic toxicities, and ocular adverse events^(1)^. Ocular side effects have been reported in patients treated with EGFR inhibitors^(3,8)^. Punctate keratitis and corneal ulcers are the most frequent signs^(3)^. Stinging, pain, and photophobia are the most frequently reported symptoms^(8)^. Ocular side effects have been revealed after treatment with MMAF inhibitors, including dry eye and photophobia as the primary side effects^(1)^. We found that ocular toxicity was limited to the ocular surface in all of our patients, with superficial keratitis the major sign and foreign-body sensation the major symptom. Both of these, in particular, began 1 week after the infusion of the treatment. Although patients occasionally presented with high grades of punctate keratitis, they surprisingly did not complain about eye pain, and corneal sensitivity was diminished using a cotton bud tip. Hence, we hypothesize that a neurotrophic component could exist^(9)^. Likewise, Parrozzani et al.^(4,5)^ observed that ABT-414 toxicity is not only directed to the corneal epithelium but also to corneal nerves. This could explain why our patients did not report any pain. Nevertheless, this is a hypothesis that will have to be studied in more detail in the future.

Although microcystic keratopathy is not fully understood, it is likely caused by the uptake of ABT-414 into limbal stem cells or transient amplification corneal cells. Evidence suggests that this toxicity might be related to general mechanisms of endocytosis rather than specific targeting of ABT-414 to activated EGFR in the cornea^(1)^.

We hypothesize that the inhibitory effects of EGFR and its synergy with MMAF inhibitor in ABT-414 treatment could provoke a greater toxic effect, damaging the corneal keratinocytes and goblet cells, finally causing ocular surface damage. Confocal microscopy or impression cytology are useful techniques for better characterization of the ocular surface^(5)^.

Some authors have asserted that rebamipide ophthalmic solution can be useful for increasing the number of goblet cells and increasing EGFR expression^(10,11)^ in the ocular surface of these patients as well as in others with an alteration of the ocular surface. This treatment may be used in cases of refractory ocular surface damage.

In another similar case series reported by Parrozzani et al.^(4,5)^, five patients had to temporarily suspend treatment and two patients had to reduce the dose of ABT-414, although no patient required a definitive withdrawal of the drug. After 4 months of follow-up, all corneal and ocular surface side effects were restored. In our case series, treatment interruption was not required in any patient, as the ocular toxicity was satisfactorily managed using topical lubrication and was restored 4 weeks after initiation of ABT-414. We believe that each situation must be carefully evaluated by both the ophthalmologist and oncologist in charge, as the decision should be based on the risks and benefits.

In conclusion, emerging molecules for the management of tumors, such as ABT-414, may produce ocular side effects. These effects are normally transient and disappear after drug discontinuation without ocular sequelae. A multidisciplinary approach, with complete ophthalmologic monitorization, is required to adequately manage these patients.
